# Tripotassium bis­(acetato-κ^2^ *O*,*O*′)(thio­cyanato-κ*N*)plumbate(II) dithio­cyanate

**DOI:** 10.1107/S1600536809021370

**Published:** 2009-06-10

**Authors:** Seik Weng Ng

**Affiliations:** a Department of Chemistry, University of Malaya, 50603 Kuala Lumpur, Malaysia

## Abstract

In the crystal structure of the title salt, K_3_[Pb(CH_3_COO)_2_(NCS)](NCS)_2_, the [Pb(CH_3_COO)_2_(NCS)]^−^ anion exists as a covalently bonded entity in which the acetate anions chelate in an anisobidentate manner. The Pb atom shows a distorted ψ-octa­hedral coordination to four acetate O atoms and one isocyanate N atom, with the stereochemically active lone pair occupying one of the six sites. When the three longer Pb⋯S inter­actions are considered, the eight-coordinate geometry is based on a dodeca­hedron. The Pb(CH_3_COO)_2_(NCS)]^−^ anion has mirror symmetry. The potassium cations connect the other constituents into a three-dimensional network through electrostatic K⋯N and K⋯S inter­actions.

## Related literature

In [K_6_Pb_6_(CH_3_CO_2_)_12_(NCS)_2_](NCS)_4_, the acetate groups link the lead(II) atoms into a chain; see: Morsali & Mahjoub (2004 ▶).
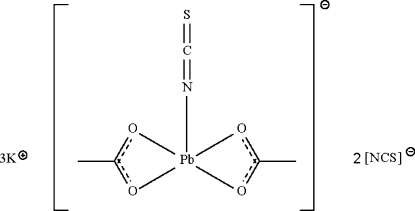


## Experimental

### 

#### Crystal data

K_3_[Pb(C_2_H_3_O_2_)_2_(NCS)](NCS)_2_
*M* *_r_* = 616.82
Monoclinic, 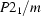

*a* = 6.1968 (1) Å
*b* = 19.2499 (3) Å
*c* = 7.6354 (1) Å
β = 106.982 (1)°
*V* = 871.10 (2) Å^3^
*Z* = 2
Mo *K*α radiation
μ = 10.77 mm^−1^
*T* = 140 K
0.25 × 0.20 × 0.08 mm

#### Data collection

Bruker SMART APEX diffractometer
Absorption correction: multi-scan (*SADABS*; Sheldrick, 1996 ▶) *T*_min_ = 0.174, *T*_max_ = 0.480 (expected range = 0.153–0.422)
6058 measured reflections
2045 independent reflections
2015 reflections with *I* > 2σ(*I*)
*R*_int_ = 0.023

#### Refinement

*R*[*F* ^2^ > 2σ(*F* ^2^)] = 0.022
*wR*(*F* ^2^) = 0.056
*S* = 1.16
2045 reflections
104 parameters
H-atom parameters constrained
Δρ_max_ = 0.81 e Å^−3^
Δρ_min_ = −2.00 e Å^−3^

### 

Data collection: *APEX2* (Bruker, 2008 ▶); cell refinement: *SAINT* (Bruker, 2008 ▶); data reduction: *SAINT*; program(s) used to solve structure: *SHELXS97* (Sheldrick, 2008 ▶); program(s) used to refine structure: *SHELXL97* (Sheldrick, 2008 ▶); molecular graphics: *X-SEED* (Barbour, 2001 ▶); software used to prepare material for publication: *publCIF* (Westrip, 2009 ▶).

## Supplementary Material

Crystal structure: contains datablocks global, I. DOI: 10.1107/S1600536809021370/tk2472sup1.cif

Structure factors: contains datablocks I. DOI: 10.1107/S1600536809021370/tk2472Isup2.hkl

Additional supplementary materials:  crystallographic information; 3D view; checkCIF report

## Figures and Tables

**Fig. 1.**
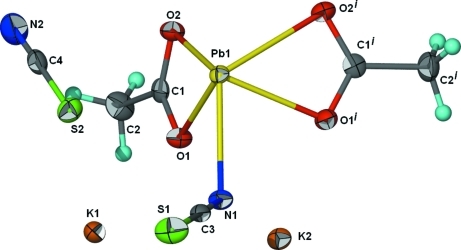
Thermal ellipsoid plot (Barbour, 2001) of a portion of the polymeric structure of K3Pb(NCS)3(CH3CO2)2 at the 70% probability level. Hydrogen atoms are drawn as spheres of arbitrary radius. Symmetry operation i: x, 1.5-y, z.

**Fig. 2.**
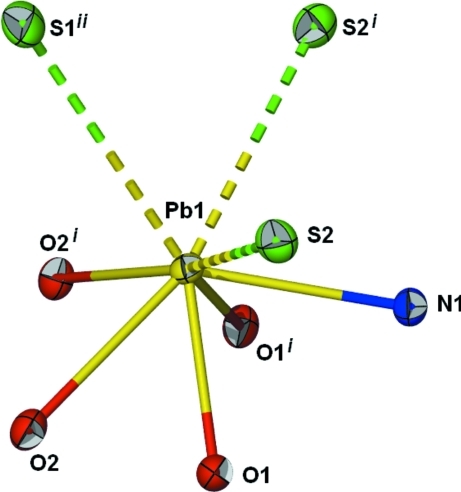
Detail showing the dodecahedral geometry of the Pb(II) atom.

## supplementary crystallographic information

### Experimental

2,4-Diamino-6-(1-piperidinyl)-2,4-pyrimidine *N*-oxide (commercial name: minoxidil) (0.5 mmol, 0.10 g), lead(II) acetate (0.5 mmol, 0.17 g) and potassium thiocyanate (1 mmol, 0.10 g) were dissolved in methanol (100 ml). The solution was concentrated to a smaller volume and this was mixed with a similar volume of chloroform. Colorless prisms separated after several days.

### Refinement

Hydrogen atoms were placed at calculated positions (C–H 0.98 Å) and were treated as riding on the carbon atom with *U*(H) set to 1.5*U*_eq_(C). The final difference Fourier map had a deep hole at 1 Å from N1.

### Crystal data

**Table:**

K_3_[Pb(C_2_H_3_O_2_)_2_(NCS)](NCS)_2_	*F*(000) = 576
*M**_r_* = 616.82	*D*_x_ = 2.352 Mg m^−^^3^
Monoclinic, *P*2_1_/*m*	Mo *K*α radiation, λ = 0.71073 Å
Hall symbol: -P 2yb	Cell parameters from 4971 reflections
*a* = 6.1968 (1) Å	θ = 2.9–28.3°
*b* = 19.2499 (3) Å	µ = 10.77 mm^−^^1^
*c* = 7.6354 (1) Å	*T* = 140 K
β = 106.982 (1)°	Block, colorless
*V* = 871.10 (2) Å^3^	0.25 × 0.20 × 0.08 mm
*Z* = 2	

### Data collection

**Table:**

Bruker SMART APEX diffractometer	2045 independent reflections
Radiation source: fine-focus sealed tube	2015 reflections with *I* > 2σ(*I*)
graphite	*R*_int_ = 0.023
ω scans	θ_max_ = 27.5°, θ_min_ = 2.1°
Absorption correction: multi-scan (*SADABS*; Sheldrick, 1996)	*h* = −7→8
*T*_min_ = 0.174, *T*_max_ = 0.480	*k* = −25→25
6058 measured reflections	*l* = −9→9

### Refinement

**Table:**

Refinement on *F*^2^	Primary atom site location: structure-invariant direct methods
Least-squares matrix: full	Secondary atom site location: difference Fourier map
*R*[*F*^2^ > 2σ(*F*^2^)] = 0.022	Hydrogen site location: inferred from neighbouring sites
*wR*(*F*^2^) = 0.056	H-atom parameters constrained
*S* = 1.16	*w* = 1/[σ^2^(*F*_o_^2^) + (0.03*P*)^2^ + 1.3438*P*] where *P* = (*F*_o_^2^ + 2*F*_c_^2^)/3
2045 reflections	(Δ/σ)_max_ = 0.001
104 parameters	Δρ_max_ = 0.81 e Å^−^^3^
0 restraints	Δρ_min_ = −2.00 e Å^−^^3^

### Fractional atomic coordinates and isotropic or equivalent isotropic displacement parameters (Å^2^)

**Table:**

	*x*	*y*	*z*	*U*_iso_*/*U*_eq_	
Pb1	0.41686 (3)	0.7500	0.47400 (2)	0.01158 (7)	
K1	0.85392 (14)	0.59423 (4)	0.42518 (12)	0.01948 (17)	
K2	0.74409 (19)	0.7500	0.09371 (15)	0.0160 (2)	
S1	1.1475 (2)	0.7500	0.8492 (2)	0.0247 (3)	
S2	0.65682 (16)	0.62677 (5)	0.76528 (14)	0.01949 (19)	
N1	0.8449 (7)	0.7500	0.4972 (6)	0.0151 (9)	
N2	0.2738 (6)	0.54336 (19)	0.6214 (6)	0.0289 (8)	
O1	0.4696 (4)	0.66654 (13)	0.2532 (4)	0.0162 (5)	
O2	0.1161 (4)	0.66405 (13)	0.2588 (4)	0.0171 (5)	
C1	0.2743 (6)	0.63941 (18)	0.2063 (5)	0.0146 (7)	
C2	0.2360 (7)	0.5741 (2)	0.0917 (6)	0.0236 (8)	
H2A	0.2283	0.5341	0.1689	0.035*	
H2B	0.3609	0.5678	0.0388	0.035*	
H2C	0.0940	0.5782	−0.0069	0.035*	
C3	0.9724 (8)	0.7500	0.6432 (7)	0.0142 (10)	
C4	0.4309 (6)	0.57822 (19)	0.6821 (5)	0.0180 (7)	

### Atomic displacement parameters (Å^2^)

**Table:**

	*U*^11^	*U*^22^	*U*^33^	*U*^12^	*U*^13^	*U*^23^
Pb1	0.01294 (11)	0.01291 (10)	0.01004 (11)	0.000	0.00516 (7)	0.000
K1	0.0164 (4)	0.0153 (4)	0.0286 (4)	0.0018 (3)	0.0095 (3)	0.0037 (3)
K2	0.0168 (5)	0.0192 (5)	0.0129 (5)	0.000	0.0058 (4)	0.000
S1	0.0184 (6)	0.0388 (8)	0.0144 (6)	0.000	0.0011 (5)	0.000
S2	0.0194 (4)	0.0185 (4)	0.0210 (5)	−0.0041 (3)	0.0065 (4)	−0.0016 (4)
N1	0.0119 (19)	0.020 (2)	0.013 (2)	0.000	0.0035 (17)	0.000
N2	0.0217 (18)	0.0232 (17)	0.036 (2)	−0.0019 (14)	0.0000 (16)	0.0014 (15)
O1	0.0136 (12)	0.0192 (12)	0.0162 (13)	−0.0004 (9)	0.0050 (10)	−0.0031 (10)
O2	0.0142 (12)	0.0194 (12)	0.0191 (13)	0.0017 (9)	0.0071 (10)	−0.0002 (10)
C1	0.0171 (16)	0.0143 (15)	0.0107 (16)	0.0017 (12)	0.0015 (13)	0.0049 (13)
C2	0.0234 (19)	0.0199 (18)	0.025 (2)	−0.0010 (15)	0.0034 (17)	−0.0079 (16)
C3	0.012 (2)	0.012 (2)	0.020 (3)	0.000	0.008 (2)	0.000
C4	0.0175 (18)	0.0142 (16)	0.0205 (19)	0.0032 (13)	0.0031 (15)	0.0034 (14)

### Geometric parameters (Å, °)

**Table:**

Pb1—O1	2.419 (3)	K2—S2^vi^	3.3788 (13)
Pb1—O1^i^	2.419 (3)	K2—S2^vii^	3.3788 (13)
Pb1—O2	2.669 (3)	S1—C3	1.630 (5)
Pb1—O2^i^	2.669 (3)	S2—C4	1.649 (4)
Pb1—N1	2.606 (4)	S2—K2^viii^	3.3788 (13)
Pb1—S1^ii^	3.713 (1)	N1—C3	1.163 (7)
Pb1—S2	3.294 (1)	N1—K1^i^	3.0520 (12)
Pb1—S2^i^	3.294 (1)	N2—C4	1.162 (5)
K1—O2^iii^	2.696 (3)	N2—K1^iv^	2.757 (4)
K1—O1	2.740 (3)	N2—K1^ii^	2.774 (4)
K1—N2^iv^	2.757 (4)	O1—C1	1.270 (4)
K1—N2^iii^	2.774 (4)	O2—C1	1.255 (5)
K1—N1	3.0520 (12)	O2—K1^ii^	2.696 (3)
K1—S2	3.2373 (13)	O2—K2^ii^	2.818 (3)
K2—O2^v^	2.818 (3)	C1—C2	1.509 (5)
K2—O2^iii^	2.818 (3)	C2—H2A	0.9800
K2—O1	2.858 (3)	C2—H2B	0.9800
K2—O1^i^	2.858 (3)	C2—H2C	0.9800
K2—N1	2.959 (5)	C3—K1^i^	3.404 (3)
			
O1—Pb1—O1^i^	83.23 (13)	O2^v^—K2—S1^ix^	143.98 (5)
O1—Pb1—N1	72.87 (9)	O2^iii^—K2—S1^ix^	143.98 (5)
O1^i^—Pb1—N1	72.87 (9)	O1—K2—S1^ix^	64.83 (6)
O1—Pb1—O2	51.04 (8)	O1^i^—K2—S1^ix^	64.83 (6)
O1^i^—Pb1—O2	101.23 (8)	N1—K2—S1^ix^	114.20 (9)
N1—Pb1—O2	123.76 (9)	S2^vi^—K2—S1^ix^	72.51 (3)
O1—Pb1—O2^i^	101.23 (8)	S2^vii^—K2—S1^ix^	72.51 (3)
O1^i^—Pb1—O2^i^	51.04 (8)	S1^vi^—K2—S1^ix^	120.08 (5)
N1—Pb1—O2^i^	123.76 (9)	C3—S1—K2^viii^	97.79 (19)
O2—Pb1—O2^i^	76.62 (11)	C3—S1—K2^x^	142.1 (2)
O2^iii^—K1—O1	94.59 (8)	K2^viii^—S1—K2^x^	120.08 (5)
O2^iii^—K1—N2^iv^	127.44 (11)	C4—S2—K1	93.34 (15)
O1—K1—N2^iv^	104.45 (9)	C4—S2—K2^viii^	128.15 (15)
O2^iii^—K1—N2^iii^	80.28 (10)	K1—S2—K2^viii^	135.65 (4)
O1—K1—N2^iii^	170.07 (10)	C3—N1—Pb1	117.3 (4)
N2^iv^—K1—N2^iii^	85.36 (11)	C3—N1—K2	151.1 (4)
O2^iii^—K1—N1	68.92 (10)	Pb1—N1—K2	91.61 (13)
O1—K1—N1	61.85 (10)	C3—N1—K1	97.45 (9)
N2^iv^—K1—N1	161.31 (12)	Pb1—N1—K1	93.42 (8)
N2^iii^—K1—N1	108.25 (11)	K2—N1—K1	79.74 (8)
O2^iii^—K1—S2	134.60 (6)	C3—N1—K1^i^	97.45 (9)
O1—K1—S2	78.90 (6)	Pb1—N1—K1^i^	93.42 (8)
N2^iv^—K1—S2	97.35 (9)	K2—N1—K1^i^	79.74 (8)
N2^iii^—K1—S2	98.58 (9)	K1—N1—K1^i^	158.52 (17)
N1—K1—S2	68.55 (9)	C4—N2—K1^iv^	141.4 (3)
O2^v^—K2—O2^iii^	71.90 (11)	C4—N2—K1^ii^	123.8 (3)
O2^v^—K2—O1	130.58 (9)	K1^iv^—N2—K1^ii^	94.64 (11)
O2^iii^—K2—O1	89.45 (8)	C1—O1—Pb1	99.1 (2)
O2^v^—K2—O1^i^	89.45 (8)	C1—O1—K1	123.3 (2)
O2^iii^—K2—O1^i^	130.58 (9)	Pb1—O1—K1	106.18 (9)
O1—K2—O1^i^	68.41 (10)	C1—O1—K2	138.4 (2)
O2^v^—K2—N1	68.78 (9)	Pb1—O1—K2	98.16 (8)
O2^iii^—K2—N1	68.78 (9)	K1—O1—K2	86.99 (8)
O1—K2—N1	61.80 (9)	C1—O2—Pb1	87.8 (2)
O1^i^—K2—N1	61.80 (9)	C1—O2—K1^ii^	126.6 (2)
O2^v^—K2—S2^vi^	132.59 (7)	Pb1—O2—K1^ii^	115.30 (10)
O2^iii^—K2—S2^vi^	81.51 (6)	C1—O2—K2^ii^	133.8 (2)
O1—K2—S2^vi^	86.29 (6)	Pb1—O2—K2^ii^	104.14 (8)
O1^i^—K2—S2^vi^	136.45 (7)	K1^ii^—O2—K2^ii^	88.66 (8)
N1—K2—S2^vi^	135.40 (2)	O2—C1—O1	121.6 (3)
O2^v^—K2—S2^vii^	81.51 (6)	O2—C1—C2	119.6 (3)
O2^iii^—K2—S2^vii^	132.59 (7)	O1—C1—C2	118.8 (3)
O1—K2—S2^vii^	136.45 (6)	C1—C2—H2A	109.5
O1^i^—K2—S2^vii^	86.29 (6)	C1—C2—H2B	109.5
N1—K2—S2^vii^	135.40 (2)	H2A—C2—H2B	109.5
S2^vi^—K2—S2^vii^	89.19 (4)	C1—C2—H2C	109.5
O2^v^—K2—S1^vi^	67.87 (6)	H2A—C2—H2C	109.5
O2^iii^—K2—S1^vi^	67.87 (6)	H2B—C2—H2C	109.5
O1—K2—S1^vi^	145.78 (5)	N1—C3—S1	179.1 (5)
O1^i^—K2—S1^vi^	145.78 (5)	N1—C3—K1^i^	62.76 (9)
N1—K2—S1^vi^	125.72 (9)	S1—C3—K1^i^	117.37 (8)
S2^vi^—K2—S1^vi^	65.92 (3)	N2—C4—S2	178.7 (4)
S2^vii^—K2—S1^vi^	65.92 (3)		
			
O2^iii^—K1—S2—C4	150.92 (15)	O1^i^—Pb1—O1—C1	−114.5 (2)
O1—K1—S2—C4	65.45 (14)	N1—Pb1—O1—C1	171.4 (2)
N2^iv^—K1—S2—C4	−37.91 (15)	O2—Pb1—O1—C1	−4.14 (19)
N2^iii^—K1—S2—C4	−124.30 (15)	O2^i^—Pb1—O1—C1	−66.5 (2)
N1—K1—S2—C4	129.38 (15)	O1^i^—Pb1—O1—K1	116.73 (7)
O2^iii^—K1—S2—K2^viii^	−9.98 (11)	N1—Pb1—O1—K1	42.61 (10)
O1—K1—S2—K2^viii^	−95.45 (7)	O2—Pb1—O1—K1	−132.90 (14)
N2^iv^—K1—S2—K2^viii^	161.19 (9)	O2^i^—Pb1—O1—K1	164.73 (9)
N2^iii^—K1—S2—K2^viii^	74.80 (9)	O1^i^—Pb1—O1—K2	27.51 (11)
N1—K1—S2—K2^viii^	−31.52 (10)	N1—Pb1—O1—K2	−46.61 (9)
O1—Pb1—N1—C3	−135.98 (7)	O2—Pb1—O1—K2	137.88 (13)
O1^i^—Pb1—N1—C3	135.98 (7)	O2^i^—Pb1—O1—K2	75.51 (9)
O2—Pb1—N1—C3	−131.78 (8)	O2^iii^—K1—O1—C1	145.2 (3)
O2^i^—Pb1—N1—C3	131.78 (8)	N2^iv^—K1—O1—C1	14.7 (3)
O1—Pb1—N1—K2	44.02 (7)	N1—K1—O1—C1	−151.6 (3)
O1^i^—Pb1—N1—K2	−44.02 (7)	S2—K1—O1—C1	−80.2 (3)
O2—Pb1—N1—K2	48.22 (8)	O2^iii^—K1—O1—Pb1	−101.92 (10)
O2^i^—Pb1—N1—K2	−48.22 (8)	N2^iv^—K1—O1—Pb1	127.50 (12)
O1—Pb1—N1—K1	−35.79 (10)	N1—K1—O1—Pb1	−38.80 (11)
O1^i^—Pb1—N1—K1	−123.83 (13)	S2—K1—O1—Pb1	32.68 (7)
O2—Pb1—N1—K1	−31.60 (15)	O2^iii^—K1—O1—K2	−4.30 (8)
O2^i^—Pb1—N1—K1	−128.03 (8)	N2^iv^—K1—O1—K2	−134.88 (10)
O1—Pb1—N1—K1^i^	123.83 (13)	N1—K1—O1—K2	58.82 (11)
O1^i^—Pb1—N1—K1^i^	35.79 (10)	S2—K1—O1—K2	130.30 (6)
O2—Pb1—N1—K1^i^	128.03 (8)	O2^v^—K2—O1—C1	158.4 (3)
O2^i^—Pb1—N1—K1^i^	31.60 (15)	O2^iii^—K2—O1—C1	−136.3 (3)
O2^v^—K2—N1—C3	−39.03 (6)	O1^i^—K2—O1—C1	89.0 (3)
O2^iii^—K2—N1—C3	39.03 (6)	N1—K2—O1—C1	157.7 (4)
O1—K2—N1—C3	140.36 (6)	S2^vi^—K2—O1—C1	−54.7 (3)
O1^i^—K2—N1—C3	−140.36 (7)	S2^vii^—K2—O1—C1	30.2 (4)
S2^vi^—K2—N1—C3	90.78 (10)	S1^vi^—K2—O1—C1	−89.4 (4)
S2^vii^—K2—N1—C3	−90.78 (10)	S1^ix^—K2—O1—C1	17.7 (3)
S1^vi^—K2—N1—C3	0.000 (2)	O2^v^—K2—O1—Pb1	44.69 (13)
S1^ix^—K2—N1—C3	180.000 (3)	O2^iii^—K2—O1—Pb1	110.02 (9)
O2^v^—K2—N1—Pb1	140.97 (6)	O1^i^—K2—O1—Pb1	−24.68 (10)
O2^iii^—K2—N1—Pb1	−140.97 (6)	N1—K2—O1—Pb1	43.94 (8)
O1—K2—N1—Pb1	−39.64 (6)	S2^vi^—K2—O1—Pb1	−168.45 (8)
O1^i^—K2—N1—Pb1	39.64 (6)	S2^vii^—K2—O1—Pb1	−83.54 (10)
S2^vi^—K2—N1—Pb1	−89.22 (10)	S1^vi^—K2—O1—Pb1	156.87 (8)
S2^vii^—K2—N1—Pb1	89.22 (10)	S1^ix^—K2—O1—Pb1	−96.05 (8)
S1^vi^—K2—N1—Pb1	180.0	O2^v^—K2—O1—K1	−61.24 (11)
S1^ix^—K2—N1—Pb1	0.0	O2^iii^—K2—O1—K1	4.10 (8)
O2^v^—K2—N1—K1	−125.85 (11)	O1^i^—K2—O1—K1	−130.60 (4)
O2^iii^—K2—N1—K1	−47.79 (8)	N1—K2—O1—K1	−61.98 (8)
O1—K2—N1—K1	53.55 (7)	S2^vi^—K2—O1—K1	85.63 (6)
O1^i^—K2—N1—K1	132.82 (11)	S2^vii^—K2—O1—K1	170.54 (5)
S2^vi^—K2—N1—K1	3.96 (16)	S1^vi^—K2—O1—K1	50.94 (14)
S2^vii^—K2—N1—K1	−177.60 (6)	S1^ix^—K2—O1—K1	158.03 (8)
S1^vi^—K2—N1—K1	−86.82 (7)	O1—Pb1—O2—C1	4.13 (19)
S1^ix^—K2—N1—K1	93.18 (7)	O1^i^—Pb1—O2—C1	75.8 (2)
O2^v^—K2—N1—K1^i^	47.79 (8)	N1—Pb1—O2—C1	−1.0 (2)
O2^iii^—K2—N1—K1^i^	125.85 (11)	O2^i^—Pb1—O2—C1	120.85 (18)
O1—K2—N1—K1^i^	−132.82 (11)	O1—Pb1—O2—K1^ii^	134.06 (15)
O1^i^—K2—N1—K1^i^	−53.55 (7)	O1^i^—Pb1—O2—K1^ii^	−154.31 (10)
S2^vi^—K2—N1—K1^i^	177.60 (6)	N1—Pb1—O2—K1^ii^	128.90 (11)
S2^vii^—K2—N1—K1^i^	−3.96 (16)	O2^i^—Pb1—O2—K1^ii^	−109.23 (8)
S1^vi^—K2—N1—K1^i^	86.82 (7)	O1—Pb1—O2—K2^ii^	−130.64 (13)
S1^ix^—K2—N1—K1^i^	−93.18 (7)	O1^i^—Pb1—O2—K2^ii^	−59.01 (10)
O2^iii^—K1—N1—C3	−100.2 (4)	N1—Pb1—O2—K2^ii^	−135.80 (10)
O1—K1—N1—C3	152.1 (4)	O2^i^—Pb1—O2—K2^ii^	−13.92 (12)
N2^iv^—K1—N1—C3	106.4 (5)	Pb1—O2—C1—O1	−7.2 (3)
N2^iii^—K1—N1—C3	−28.7 (4)	K1^ii^—O2—C1—O1	−127.5 (3)
S2—K1—N1—C3	63.5 (3)	K2^ii^—O2—C1—O1	100.3 (4)
O2^iii^—K1—N1—Pb1	141.70 (14)	Pb1—O2—C1—C2	170.0 (3)
O1—K1—N1—Pb1	34.03 (9)	K1^ii^—O2—C1—C2	49.7 (4)
N2^iv^—K1—N1—Pb1	−11.7 (4)	K2^ii^—O2—C1—C2	−82.5 (4)
N2^iii^—K1—N1—Pb1	−146.82 (12)	Pb1—O1—C1—O2	8.0 (4)
S2—K1—N1—Pb1	−54.57 (9)	K1—O1—C1—O2	124.4 (3)
O2^iii^—K1—N1—K2	50.68 (9)	K2—O1—C1—O2	−105.3 (4)
O1—K1—N1—K2	−56.99 (9)	Pb1—O1—C1—C2	−169.2 (3)
N2^iv^—K1—N1—K2	−102.7 (4)	K1—O1—C1—C2	−52.9 (4)
N2^iii^—K1—N1—K2	122.16 (11)	K2—O1—C1—C2	77.4 (4)
S2—K1—N1—K2	−145.59 (10)	Pb1—N1—C3—K1^i^	−97.76 (12)
O2^iii^—K1—N1—K1^i^	33.4 (4)	K2—N1—C3—K1^i^	82.24 (12)
O1—K1—N1—K1^i^	−74.3 (4)	K1—N1—C3—K1^i^	164.5 (2)
N2^iv^—K1—N1—K1^i^	−120.0 (5)	K2^viii^—S1—C3—K1^i^	97.27 (19)
N2^iii^—K1—N1—K1^i^	104.8 (4)	K2^x^—S1—C3—K1^i^	−82.73 (19)
S2—K1—N1—K1^i^	−162.9 (5)		

Symmetry codes: (i) *x*, −*y*+3/2, *z*; (ii) *x*−1, *y*, *z*; (iii) *x*+1, *y*, *z*; (iv) −*x*+1, −*y*+1, −*z*+1; (v) *x*+1, −*y*+3/2, *z*; (vi) *x*, *y*, *z*−1; (vii) *x*, −*y*+3/2, *z*−1; (viii) *x*, *y*, *z*+1; (ix) *x*−1, *y*, *z*−1; (x) *x*+1, *y*, *z*+1.
